# A Combined Self-Consistent Method to Estimate the Effective Properties of Polypropylene/Calcium Carbonate Composites

**DOI:** 10.3390/polym10010101

**Published:** 2018-01-21

**Authors:** Zhongqiang Xiong, Shaorong Lu, Junkun Liu, Guangsheng Lv, Yuqi Li, Jinhong Yu

**Affiliations:** 1 Key Laboratory of New Processing Technology for Nonferrous Metals and Materials, Ministry of Education, College of Materials Science and Engineering, Guilin University of Technology, Guilin 541004, China; xiongzhongq@163.com (Z.X.); a2692742@163.com (J.L.); a1053874264@163.com (G.L.); 2 Key Laboratory of Marine Materials and Related Technologies, Zhejiang Key Laboratory of Marine Materials and Protective Technologies, Ningbo Institute of Material Technology & Engineering, Chinese Academy of Sciences, Ningbo 315201, China

**Keywords:** micromechanics, anisotropy, polypropylene composites, hyper-dispersant

## Abstract

In this work, trying to avoid difficulty of application due to the irregular filler shapes in experiments, self-consistent and differential self-consistent methods were combined to obtain a decoupled equation. The combined method suggests a tenor ***γ*** independent of filler-contents being an important connection between high and low filler-contents. On one hand, the constant parameter can be calculated by Eshelby’s inclusion theory or the Mori–Tanaka method to predict effective properties of composites coinciding with its hypothesis. On the other hand, the parameter can be calculated with several experimental results to estimate the effective properties of prepared composites of other different contents. In addition, an evaluation index $\sigma_{f}^{\prime }$ of the interactional strength between matrix and fillers is proposed based on experiments. In experiments, a hyper-dispersant was synthesized to prepare polypropylene/calcium carbonate (PP/CaCO_3_) composites up to 70 wt % of filler-content with dispersion, whose dosage was only 5 wt % of the CaCO_3_ contents. Based on several verifications, it is hoped that the combined self-consistent method is valid for other two-phase composites in experiments with the same application progress as in this work.

## 1. Introduction

As a consequence of component coordination, composites have come to have wider applications. It is significant that a composite can be designed with a satisfied coordination according to the special situation [1,2,3]. To realize the relation between structure and properties, composite mechanics has been extensively investigated, not only in terms of preparation and characterization in engineering, but also modeling and simulation in theory.

In material engineering, there are many experiments that are executed regularly to find the best composite with the optimized dosage and processing conditions. The dispersion and compatibility of fillers in a matrix are the most important factors impacting effective properties of composites. Therefore, the dispersant, coupling agent and surface modifier are used for improving the dispersion and strength of interphase among different components [4,5,6,7,8,9,10]. Different agents need to be designed in special situations. The toughening and debonding mechanisms [11,12,13] between the structures and properties are investigated with the conjecture of strong ligaments [13,14] and micro-cracks [15,16], respectively. Regardless of the optimized processing conditions or improved dispersion and compatibility that are used, efforts should try to obtain a specific microstructure. Meanwhile, there are many methods of microstructure analysis by measurement techniques to check dispersion or morphology of fillers [17,18,19]. Therefore, how the microstructure influences the properties of composites is the key to designing an excellent composite of given components as well as to increase efficiency of experiments to save resources.

In theory of micromechanics, the influence of microstructures is characterized by methods of homogenization [20,21] based on a strong interfacial interaction hypothesis, such as the self-consistent [20,22], differential self-consistent [23], Mori–Tanaka method [24,25,26,27] and generalized self-consistent [28,29] methods. The microstructure information [30,31,32,33], such as shape, orientation and distribution, of components are reflected by different localized coefficients. Owing to the fact that microstructure information is difficult to know overall, specially in complicated situations, the restrictions of effective properties of composites are revealed by the principle of minimum potential and complementary energy; for instance [34,35], Reuss lower-bound and Voigt upper-bound. The general estimation method of the effective stiffness of composites is Hashin–Shtrikman calculus of variations [20,36,37]. In a simple way, most of the properties of composites are estimated by the rule of mixtures [20,38] and revised by the hybrid effect [39,40] for special conditions. However, several perfect assumptions of the filler shapes and strength of interphase are far away from engineering conditions and the numerical iteration of self-consistent methods increases the difficulty to analyze the properties of materials.

Although the influence of microstructures on the effective properties of composites has been uncovered in theory, situations such as irregular filler shapes or poor strength of interphase in engineering are so complicated that the effective properties of composites are hard to predict. In this paper, the strain localization relations of both self-consistent and differential self-consistent methods were regarded as identity in the same two-phase material as shown in Section 2. Hence, two self-consistent methods can be combined to estimate the effective properties of a composite more conveniently even for the experimental conditions. The solutions of the combined self-consistent methods were discussed in cases of both anisotropy and isotropy as shown in Section 3. Especially, a simplified isotropic mixture model of the combined method is applied in experiments as shown in Section 4, which is the Voigt model when *γ_v_ = 1* or the Reuss model when *γ_v_ = E_m_/E_f_*. In addition, an evaluation index $\sigma_{f}^{\prime }$ is proposed for evaluating the interactional strength between matrix and fillers. In experiments, a new hyper-dispersant was synthesized to prepare PP/CaCO_3_ composites up to 70 wt % of filler-content that is well-dispersed. Verified by comparing with the Mori–Tanaka method, the FEA confirmed SP model and experiments, it is hoped that the combined self-consistent method is valid for other two-phase composites in experiments, especially for other particle reinforced composites. For a valid application of the combined method, the strict processing and careful characterization of a composite are necessary.

## 2. Combined Self-Consistent Method 

**Figure 1:**
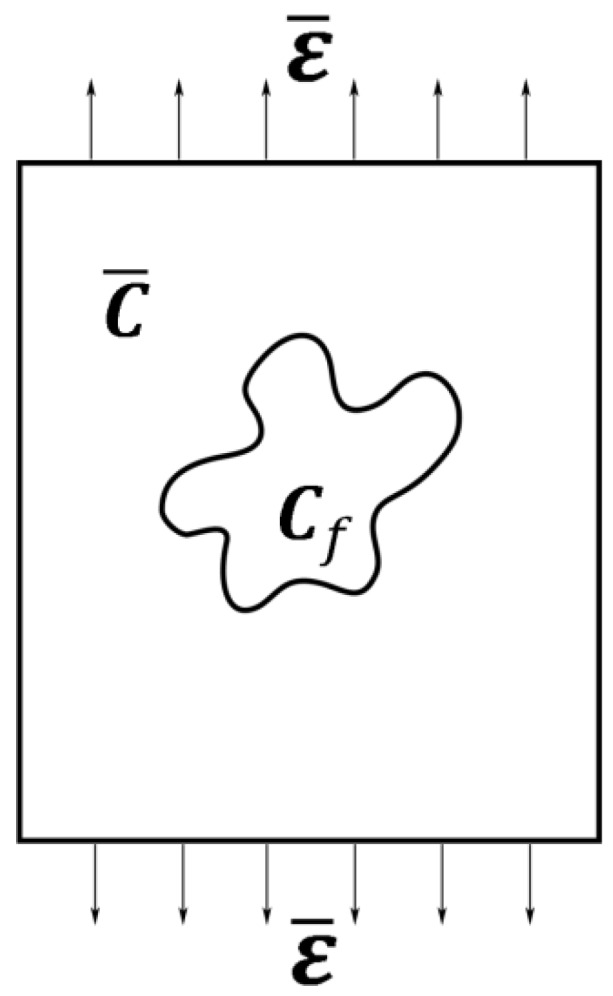
The single-filler transition of self-consistent approach in the RVE.

The different dispositions of strain localization relation result in different estimation methods. The strain localization relation in the representative volume element (RVE) is written as

$${\langle \varepsilon \rangle }_{f}={\bar{B}}_{f}∶\bar{\varepsilon },$$

where $\bar{\varepsilon }$ is external strain on boundary of composites; ${\langle \varepsilon \rangle }_{f}$ is average strain in a filler. The self-consistent method proposes that fillers put into a matrix which is regarded as a composite with a pending property as shown in Figure 1. Thus, tensor ${\bar{B}}_{f}$ depends on the pending property of a composite and also the shape or orientation of fillers. Meanwhile, the effective stiffness of a two-phase composite is characterized by the self-consistent method with $\bar{C}=C_{m}+v_{f}\left(C_{f}-C_{m}\right)∶{\bar{B}}_{f}$ [20,22]. Hence, the pending tensor ${\bar{B}}_{f}$ is written as

(1) $${\bar{B}}_{f}=v_{f}^{-1}{\left(C_{f}-C_{m}\right)}^{-1}∶\left(\bar{C}-C_{m}\right),$$

where the superscript −1 denotes the inverse operation of a tensor; $C_{m}$, $C_{f}$ and $\bar{C}$ are the stiffness tensor of matrix, filler and composite, respectively; $v_{f}$ is volume fraction of fillers in the composite.

On the other hand, there is a method of differential self-consistent [23], which the matrix is replaced gradually by fillers to construct the final composite. The incremental removal-replacement shows the homogenization relation at stage of content $v_{f}$ as

(2) $$\bar{C}\left(v_{f}+dv_{f}\right)=\bar{C}\left(v_{f}\right)+\frac{dv_{f}}{1-v_{f}}\left[C_{f}-\bar{C}\left(v_{f}\right)\right]∶{\bar{B}}_{f},$$

where the ${\bar{B}}_{f}$ depends exactly on the pending property of composite at stage of content $v_{f}$. Therefore, substituting Equation (1) into Equation (2), the combined self-consistent equation for two-phase composite is obtained

(3) $$\left(1-v_{f}\right)v_{f}\frac{dC_{f-v}}{dv_{f}}=C_{f-v}∶C_{f-m}^{-1}∶C_{f-v}-C_{f-v},$$

where $C_{f-v}=C_{f}-\bar{C}$ and $C_{f-m}^{-1}={\left(C_{f}-C_{m}\right)}^{-1}$. The boundary condition ${C_{f-v}|}_{v_{f}=0}=C_{f}-C_{m}$ or ${C_{f-v}|}_{v_{f}=1}=0$ is inherent in Equation (3). The effective stiffness tensor of a composite $\bar{C}$ is determined by Equation (3). Especially, this method is good at calculating the situation that ${\bar{B}}_{f}$ can be regarded as a state function of filler-content owing to the identical strain localization relation of both self-consistent methods. 

## 3. Discussion of the Combined Self-Consistent Method 

### 3.1. Solutions of the Combined Equation

#### 3.1.1. The Anisotropic Case

For a general discussion to the combined approach, Equation (3) is solved directly in this section, which can be linearized by the inverse tensor $C_{f-v}^{-1}$ at both sides of equation simultaneously. Noting that $C_{f-v}^{-1}∶dC_{f-v}=-\left(dC_{f-v}^{-1}\right)∶C_{f-v}$, Equation (3) is transformed as

$$\left(1-v_{f}\right)v_{f}\frac{dC_{f-v}^{-1}}{dv_{f}}=C_{f-v}^{-1}-C_{f-m}^{-1}.$$

Therefore, the equation becomes a decoupled first-order linear tensor equation. The general solution is

(4) $$C_{f-v}^{-1}=C_{f-m}^{-1}+\frac{v_{f}}{1-v_{f}}\alpha ,$$

where α is a constant tensor where each element is greater than zero. Owing to the flexibility or stiffness tensor is Voigt symmetric, there is the same symmetry for α, i.e., $\alpha_{ijkl}=\alpha_{jikl}=\alpha_{ijlk}=\alpha_{klij}$, which are just 21 independent constants in the three-dimensional case. Alternatively, Equation (4) is converted into

(5) $$\bar{C}=C_{f}-\left(C_{f}-C_{m}\right)∶{\left(I+\frac{v_{f}}{1-v_{f}}\gamma \right)}^{-1},$$

where the dimensionless parameter $\gamma =\alpha ∶\left(C_{f}-C_{m}\right)$ and I is the unit fourth-order tensor, $I_{ijkl}=\left(\delta_{ik}\delta_{jl}+\delta_{il}\delta_{jk}\right)/2$. The constant tensor γ is more significant than ${\bar{B}}_{f}$ because γ does not associate with filler-contents anymore but still retain the information of different microstructures. This is an important connection between high and low filler-contents, while low filler-contents case is well-known by Eshelby's equivalent inclusion theory or the Mori–Tanaka method. For anisotropic two-phase composites, the tensor ${\bar{B}}_{f}$ can be calculated by the constant tensor γ with different filler-content $v_{f}$

(6) $${\bar{B}}_{f}=\frac{1}{v_{f}}\left[I-{\left(I+\frac{v_{f}}{1-v_{f}}\gamma \right)}^{-1}\right],$$

which is the combination of Equations (1) and (5). The explicit function avoids the numerical iteration of self-consistent methods. Hereto, the only assumption is the identical strain localization tensor of both self-consistent methods in the same materials for the above discussion. 

For some special cases, when γ=I, Equation (5) can be simplified into

$$\bar{C}=\left(1-v_{f}\right)C_{m}+v_{f}C_{f},$$

and when $\gamma =C_{f}^{-1}∶C_{m}$, Equation (5) can be simplified into

$${\bar{C}}^{-1}=\left(1-v_{f}\right)C_{m}^{-1}+v_{f}C_{f}^{-1}.$$

#### 3.1.2. The Isotropic Case

When a two-phase composite can be treated as a macroscopic isotropic material whose fillers are regarded as different spheres distribution or short-fibers without orientation in statistics, the stiffness tensors of composite and components are isotropic, i.e., $C_{ijkl}=\lambda \delta_{ij}\delta_{kl}+\mu \left(\delta_{ik}\delta_{jl}+\delta_{il}\delta_{jk}\right)$ as well as $\gamma_{ijkl}=\gamma_{1}\delta_{ij}\delta_{kl}+\gamma_{2}\left(\delta_{ik}\delta_{jl}+\delta_{il}\delta_{jk}\right)$. Hence, the general solution about $\bar{\lambda },\;\bar{\mu }$ can be obtained from Equation (5)

(7) $$\{\begin{array}{c} \bar{\lambda }=\frac{\left[\left(3\gamma_{1}+2\gamma_{2}\right)\lambda_{f}-\lambda_{m}\right]v_{f}+\lambda_{m}+\frac{2\gamma_{1}\left(1-v_{f}\right)v_{f}}{\left(2\gamma_{2}-1\right)v_{f}+1}\left(\mu_{f}-\mu_{m}\right)}{\left(3\gamma_{1}+2\gamma_{2}-1\right)v_{f}+1}\\ \bar{\mu }=\frac{\left(2\gamma_{2}\mu_{f}-\mu_{m}\right)v_{f}+\mu_{m}}{\left(2\gamma_{2}-1\right)v_{f}+1} \end{array},$$

where λ, μ are Lame constants; the labels m, f and bar denote matrix, filler and composite, respectively. If the definitions of effective modulus $\bar{E}$ and effective Poisson’s ratio $\bar{\eta }$ are allowed, we can further discuss

$$\bar{E}=\frac{\bar{\lambda }\bar{\mu }}{\bar{\lambda }+\bar{\mu }}+2\bar{\mu },\;\bar{\eta }=\frac{\bar{\lambda }}{2\left(\bar{\lambda }+\bar{\mu }\right)}.$$

To an excellent approximation, if the difference of Poisson’s ratios of components and composite is small enough, a simple solution of $\bar{E}$ can be obtained

(8) $$\bar{E}/E_{m}=\frac{\left(\gamma_{v}E_{f}/E_{m}-1\right)v_{f}+1}{\left(\gamma_{v}-1\right)v_{f}+1}=\frac{\left(\gamma_{w}E_{f}/E_{m}-1\right)w_{f}+1}{\left(\gamma_{w}-1\right)w_{f}+1},$$

where $E_{m}$, $E_{f}$ and $\bar{E}$ are the modulus of matrix, filler and composite, respectively. Dimensionless parameter $\gamma_{v}$ is a constant constrained by $\gamma_{v}>0$. Combining with the relation between mass fraction $w_{f}$ and volume fraction $v_{f}$, $v_{f}^{-1}-1=\left(\rho_{f}/\rho_{m}\right)\left(w_{f}^{-1}-1\right)$, we can also obtain a relation of composites’ modulus associated with the fillers mass fraction as shown in Equation (8), where $\gamma_{w}=\gamma_{v}\rho_{m}/\rho_{f}$. $\rho_{m}$ and $\rho_{f}$ are the density of matrix and filler, respectively. Especially, the isotropic mixture model Equation (8) can be simplified into

$$\{\begin{array}{c} \bar{E}=\left(1-v_{f}\right)E_{m}+v_{f}E_{f}\;\mathrm{when}\;\gamma_{v}=1\;\\ {\bar{E}}^{-1}=\left(1-v_{f}\right)E_{m}^{-1}+v_{f}E_{f}^{-1}\;\mathrm{when}\;\gamma_{v}=E_{m}/E_{f} \end{array}.$$

Thus, the isotropic mixture model is the Voigt model (in-parallel model) when $\gamma_{v}=1$ or the Reuss model (in-series model) when $\gamma_{v}=E_{m}/E_{f}$. Meanwhile, the Voigt model and Reuss model are the upper-bound and lower-bound of the effective modulus of a composite, respectively [34,35]. Hence, the value of $\gamma_{v}$ is restricted in $E_{m}/E_{f}\leq \gamma_{v}\leq 1$.

### 3.2. Verifications of These Solutions

#### 3.2.1. Comparison with the Mori-Tanaka Method

The Mori-Tanaka method considers the interaction among fillers. The strain localization tensor of a two-phase composite is written as ${\bar{B}}_{MT}=\gamma_{0}∶{\left[\left(1-v_{f}\right)I+v_{f}\gamma_{0}\right]}^{-1}$ [27,31] with

(9) $$\gamma_{0}={\left[I+E_{s}∶C_{m}^{-1}∶\left(C_{f}-C_{m}\right)\right]}^{-1},$$

where $E_{s}$ is the Eshelby’s tensor associated with filler shapes, which has the explicit formula only for the regular filler shape [41]. $\gamma_{0}$ is determined by the properties of components and the way of combination. Moreover, this strain localization tensor can be presented as

$$\begin{array}{c} I=\left[\left(1-v_{f}\right)I+v_{f}\gamma_{0}\right]∶{\left[\left(1-v_{f}\right)I+v_{f}\gamma_{0}\right]}^{-1}\\ \;=\left(1-v_{f}\right){\left[\left(1-v_{f}\right)I+v_{f}\gamma_{0}\right]}^{-1}+v_{f}{\bar{B}}_{MT}\\ \;={\left(I+\frac{v_{f}}{1-v_{f}}\gamma_{0}\right)}^{-1}+v_{f}{\bar{B}}_{MT}. \end{array}$$

Hence the strain localization tensor of Mori-Tanaka method of two-phase composites is

$${\bar{B}}_{MT}=\frac{1}{v_{f}}\left[I-{\left(I+\frac{v_{f}}{1-v_{f}}\gamma_{0}\right)}^{-1}\right].$$

The form of this formula is similar to Equation (6). It implies a nice connection between the combined approach and Mori–Tanaka method. But the set of $\gamma_{0}$ is only a subset of γ. The parameter γ comes from the process of combined equation without any restriction but just a constant tensor related to the properties of raw materials. Therefore, the combined approach suggests that $\gamma_{0}$ can be shifted to another constant tensor γ even if there is not Equation (9) anymore. These indicate more widely applications of the combined method. More accurate estimation depends on how to evaluate the parameter γ. 

For discussing the connection between the combined self-consistent method and Mori–Tanaka method, the brackets of Equation (6) are expanded and we get a simple relation $\gamma =\underset{v_{f}\to 0}{\lim}{\bar{B}}_{f}$. In a significant special case that single ellipsoidal filler embeds in an infinite matrix, the Eshelby equivalent inclusion theory shows

$$\gamma =\underset{v_{f}\to 0}{\lim}{\bar{B}}_{f}=\gamma_{0}.$$

It should be noticed that the physical meaning of the limit process is ambiguous. For instance, if ${\bar{B}}_{f}$ is continuous at $v_{f}=0$, thus $\gamma =\underset{v_{f}\to 0}{\lim}{\bar{B}}_{f}={\bar{B}}_{f}\left(0\right)=I$. This is the Voigt upper-bound as shown in special case of Section 3.1.1. If there is a single ellipsoidal filler in the infinite matrix, the conclusion is Equation (9). If there are several fillers close enough in a large matrix that the interaction of fillers is appreciable, or say aggregation, the meaning of γ is not obvious, but it also coincides with the limit process. Hence, the relation, $\gamma =\underset{v_{f}\to 0}{\lim}{\bar{B}}_{f}$, is just a kind of special situation, which is convenient but unnecessary for a theoretic discussion. Ultimately, no matter how many filler-contents are, γ should be determined by Equation (6) if ${\bar{B}}_{f}$ is known first.

#### 3.2.2. Comparison with the SP Model

**Figure 2:**
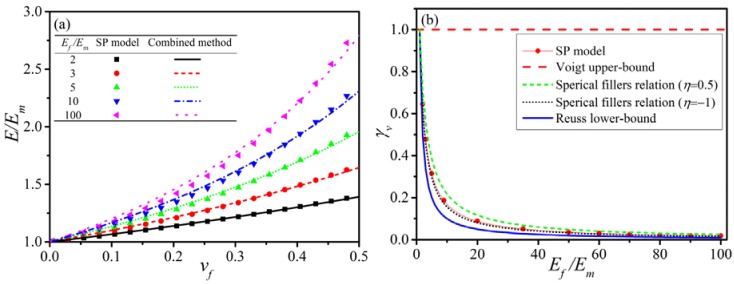
(**a**) Comparison between SP model (FEA) and Equation (8); (**b**) The meaning of $\gamma_{v}$ in special cases.

J.F. Tan et al. [38] establish a series-parallel mixture model (SP model) for particle reinforced composites. For the spherical fillers, the relation of $\gamma_{v}$ related to the properties of components can be simplified from Equation (9) with the spherical hypothesis in $E_{s}$

(10) $$\gamma_{v}=\frac{15\left(1-\eta \right)}{\left(8-10\eta \right)E_{f}/E_{m}+\left(7-5\eta \right)},$$

where $\eta \equiv \left(\bar{\eta }\approx \eta_{m}\approx \eta_{f}\right)$ is Poisson’s ratio restricted in −1<η<0.5 only for isotropic materials. Similar to the fitted SP model, Equation (10) is regarded as a fitting relation with fitted parameter η=0.01, which is allowed when $\gamma_{v}$ is constant for volume fraction $v_{f}$ as discussed in Section 3.2.1. Comparing the isotropic mixture model Equation (8) and its parameter Equation (10) with the SP model that is confirmed by the finite element analysis (FEA), the results are shown in Figure 2a,b. It indicates the adaptability of the isotropic mixture model Equation (8) to the particle reinforced composites. If Poisson’s ratios of the matrix and filler are considerably different, the more accurate discussion should rely on Equation (7).

## 4. Applications in Experiments

In theory, there is often a hypothesis of strong interfacial interaction for the convenience of the FEA calculation or establishment of homogenization methods. The strong interfacial interaction of given components insures the best practical level of prepared composites. Thus, our experiments are facing two main big problems, a better dispersion and compatibility of filler in matrix to close the theoretical hypothesis. Therefore, a hyper-dispersant was designed for the PP/CaCO_3_ composite. And we checked the dispersion of CaCO_3_ with scanning electron microscopy (SEM).

### 4.1. Experiments

#### 4.1.1. Synthesis of Polyethylene Polyamine Hyper-Dispersant: Polyethylene Polyamine Grafted Poly(12-Hydroxy Stearate) (PEPA-*g*-PHS)

**Figure 3:**
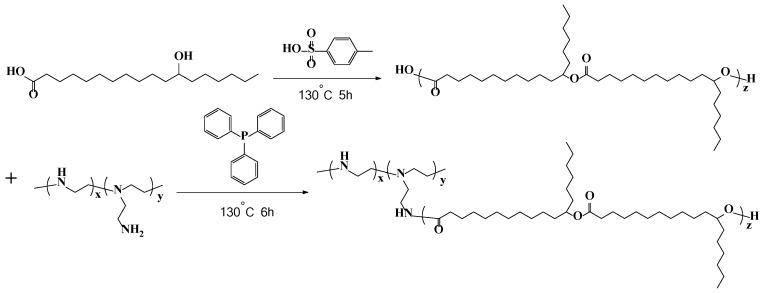
A synthesis route of PEPA-*g*-PHS.

(i) 12-hydroxy stearic acid (300 g) and *p*-toluene sulfonic acid (3.75 g) were added into a 500 mL three-neck round bottomed flask with a stirrer and thermometer. Heated to 130 °C under continuous vacuuming and stirring after reactants melt completely, the reaction was held for 5 h. Products were dried in an oven at 60 °C until constant weight after finishing the reaction. (ii) the products (200 g) of (i), polyethylene polyamine (9.4 g) and triphenylphosphine (2.6 g) were added into a 500 mL three-necked round bottomed flask with a stirrer and thermometer. Heated to 130 °C, the reactants were stirred under an atmosphere of N_2_ for 6 h. Finally, products were preserved. The typical procedure of preparing PEPA-*g*-PHS was described in Figure 3. All reagents and chemicals were used without further purification. The characterization of FT-IR, Optical photographs and the Tu-4 cup viscosity of PEPA-*g*-PHS are shown in Appendix A.

#### 4.1.2. Preparation of the PP/CaCO_3_ Composites

PP (PPH-T03, Standard: Q/SHPRD253-2009, with a melt flow rate (MFR) of 1.31 g·(10 min)^−1^ (at 200 °C, 10 kg) and a density of 0.88 g·cm^−3^) and CaCO_3_ (Light calcium carbonate of edible grade, Standard: GB1898-2007, with 1500 mesh and a density of 2.47 g·cm^−3^) used in this work were commercially available. Before preparation of composites, all the raw materials were dried in an oven at 60 °C. In this work, the addition of PEPA-*g*-PHS was 5 wt % of the CaCO_3_ contents to modify CaCO_3_ particles. The contents of modified CaCO_3_ were $w_{f}$ varied from 0 to 70 wt % in PP/CaCO_3_ composites, i.e., contents of PP were $1-w_{f}$. The modified CaCO_3_ and PP were processed through a co-rotating twin-screw extruder (TLE16-4). The melt temperature was set to 200 °C, and the screw speed was maintained at 65 rpm. The mixture was pelleted and dried. Then the pellets were shaped by the injection molding machine (TW-25V-1S) with 200 °C to obtain the standard specimen, which the mold temperature was maintained at room temperature.

#### 4.1.3. Test Procedure and Characterization of PP/CaCO_3_ Composites

Mechanical properties of the composites were measured by uniaxial tension and three-point bending with an electronic universal testing machine (SUNS UTM14483) according to the tensile test standard GB/T 1040.2-2006 and the bending test standard GB/T 9341-2008, respectively. The tensile speed was 20 mm·min^−1^ and the bending speed was 2 mm·min^−1^. All tests of specimens were carried out at room temperature and all experimental data were processed in Origin 9.0.0 (OriginLab, Northampton, MA, USA) and MTLAB R2012b (MathWorks, Natick, MA, USA) in this paper.

**Figure 4:**
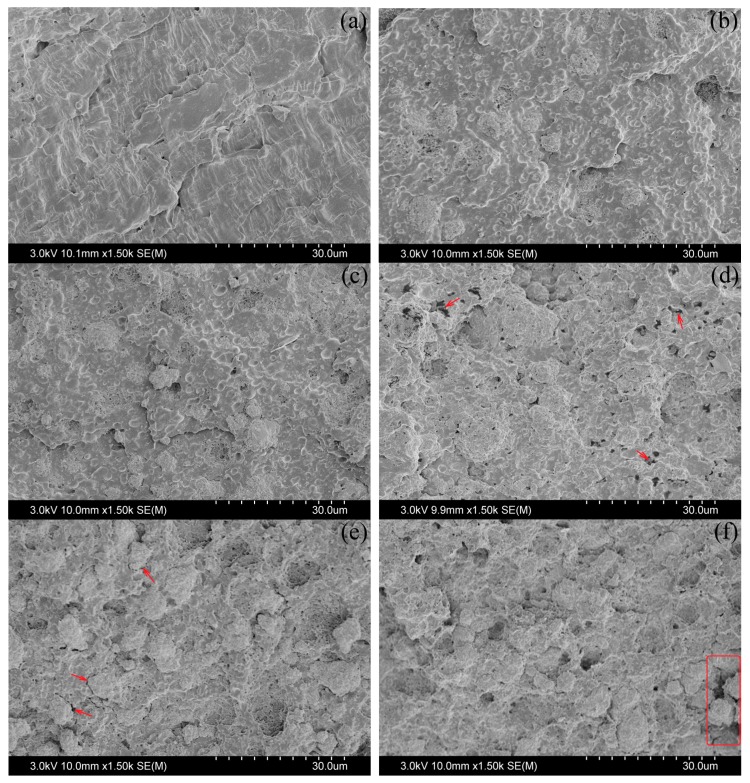
SEM images of fracture surfaces of PP composites with different CaCO_3_ contents: (**a**) 0 wt %; (**b**) 15 wt %; (**c**) 30 wt %; (**d**) 50 wt %; (**e**) 60 wt %; (**f**) 70 wt %.

Field emission scanning electron microscopy (FE-SEM, JSM-6701F, JEOL, Tokyo, Japan) was used to characterize the impact-fractured surface morphologies of specimens with an accelerating voltage of 3.0 kV. The impact fracture surfaces were coated with a thin layer of gold to avoid the accumulation of charge. As shown in Figure 4, there is more rough fracture surface with higher filler-contents and more particles that can be seen. The micron-level particles separate from each other, which shows dispersion of CaCO_3_ particles even for higher filler-contents except a few aggregations shown in the box of Figure 4f.

### 4.2. Applications of the Combined Self-Consistent Method

#### 4.2.1. The Variation Tendency of Moduli

Although the influences of microstructures on the effective properties of composites have been uncovered in a lot of simple conditions, the effective properties of composites in engineering are still hard to predict. In experiments, the hypothesis is often violated even if the simplest particles are irregular as shown in Figure 4. If there are complex shapes, Equation (10) is incorrect and not a simple formula anymore but $\gamma_{v}$ is still a constant based on the combined self-consistent method. Therefore, the certain expression of γ ($\gamma_{v}$) with many hypotheses may not be relevant in experiments; but we know that γ ($\gamma_{v}$) is only related to the properties of both matrix and filler including parameters of the shape. Thus, the γ ($\gamma_{v}$) is regarded as a whole determined by several experimental data. And then we estimate the effective properties of composites of other $v_{f}$ based on the determined parameter γ ($\gamma_{v}$). This progress is applied to both tensile and bending moduli because they are no essential differences in theory. For instance, both moduli are equal to each other in theoretical conditions but affected by the different test conditions in experiments [42].

For instance of an application, we use the simplest Equation (8) for an isotropic material. If we selected the filler and matrix according to the basic condition of the formula, the two values of $E_{f}$ and $E_{m}$ were certain. These two materials were strictly processed into a composite with the volume fraction $v_{f}=v_{0}$. Testing the composite based on the testing standard, we obtained an experimental value of its modulus, $\bar{E}={\bar{E}}_{0}$. Hence, the value of $\gamma_{v}$ was obtained by a single valid experiment

$$\gamma_{v}=\left(\frac{1-v_{0}}{v_{0}}\right)\left(\frac{{\bar{E}}_{0}-E_{m}}{E_{f}-{\bar{E}}_{0}}\right),$$

then substituted back into Equation (8). Hence the effective properties of different volume fraction composites were evaluated. More accurately, we can carry out more valid experiments whether the volume fraction is the same or not, or utilize more accurate model such as Equation (7). To fit these experimental data, the parameters of the model are determined. It is recommended by executing different volume fraction experiments with uniform distribution. For a discussion of anisotropy, Equation (5) should be employed. The γ can be determined as long as there is at least one experimental datum of $\left(v_{f},\;\bar{C}\right)$ to calculate. The fourth-order tensor is often converted into a square matrix according to a certain procedure (Voigt notation) for exhibiting the anisotropic material constants in engineering [43]. It is worthwhile to note that all of these discussions are based on the dispersion and compatibility of filler in matrix, which is the guarantee of valid application of the combined method. Hence, the strict processing and careful characterization of a composite are necessary in experiments.

**Figure 5:**
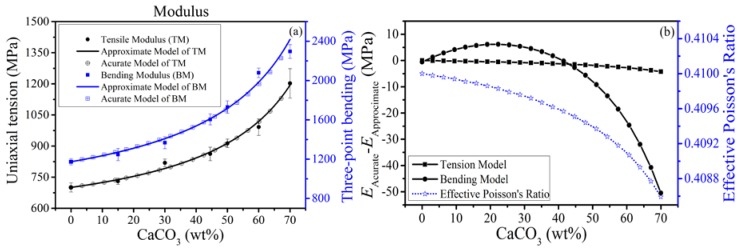
(**a**) Variation tendencies of moduli; (**b**) comparison of two isotropic models and the variation tendency of effective Poisson’s ratio.

The presence of CaCO_3_ enhances the modulus of PP in terms of both tension and bending as is shown in Figure 5a. It presents obvious relations, that the moduli of both tension and bending increase with contents of modified CaCO_3_ in experiment. As shown in SEM images in Figure 4e,f, the CaCO_3_ particles are irregular and distributed. For convenience, the PP/CaCO_3_ composites were treated as isotropic materials, where the shape of CaCO_3_ particles is random without orientation in statistics. Hence, according to the discussion of Section 3.1.2, Equation (8) is employed as being an approximate model when the differences of Poisson’s ratios of both components and composite are ignored. For a more accurate model in Equation (7), the differences of Poisson’s ratios of components are considered and the common values, $\eta_{m}=0.41,\;\eta_{f}=0.30$, are used. 

As shown in Figure 5a, experiments conform well with both isotropic models. The fitting results of both isotropic models are shown in Table 1. According to Figure 5a,b, the difference of estimated effective modulus of the PP/CaCO_3_ composites is small (~3.83%) between the accurate and approximate models. The approximate model is much simpler but the accurate model can discuss the change tendency of effective Poisson’s ratios whose difference is ignored by the approximate model. Experiments conform well to the theory, which is an indirect demonstration about a well-dispersion of CaCO_3_ benefited by the PEPA-*g*-PHS; it is also demonstrated by the elongation at break and typical stress-strain curves of different contents PP/CaCO_3_ composites as shown in Appendix B.

#### 4.2.2. The Variation Tendency of Yield Strength

**Figure 6:**
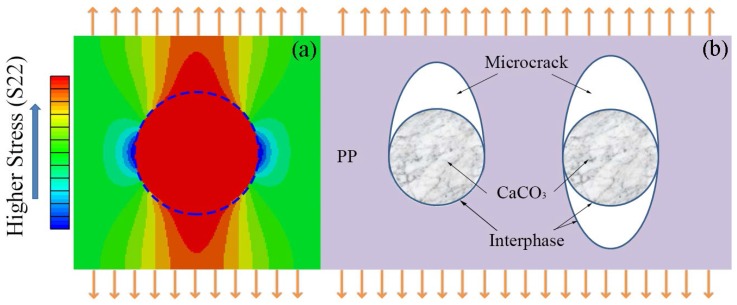
(**a**) Stress distribution [38]; (**b**) Two types of micro-crack at interphase in imagining.

**Figure 7:**
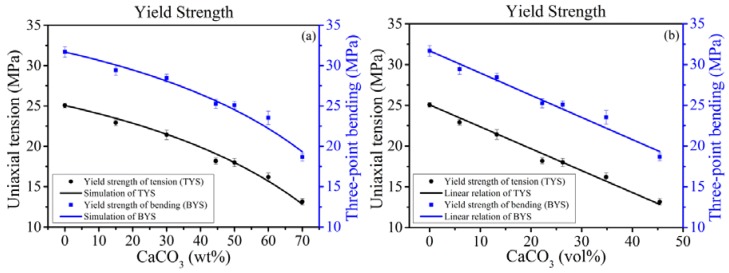
Variation tendencies of yield strengths with (**a**) mass fraction or (**b**) volume fraction.

The spherical filler suffers most of stresses according to Figure 6a of FEA with a considerably fine mash when there is a strong interfacial interaction [38]. It should have a well-reinforced effect benefited by the higher yield strength $\sigma_{f}$ of CaCO_3_. However, the yield strengths of both tension and bending decrease with contents of modified CaCO_3_ in experiments as shown in Figure 7. 

It is distinct from the modulus of a material that the strength is displayed at yield point where the composite structure has been changed. Because there is often a poor interphase strength in our experiments, the PEPA-*g*-PHS is just adsorbed on the surface of CaCO_3_ particles and relied on the intermolecular forces. The adsorption is not strong enough to sustain tension stress. Thus, the micro-crack is generated at a two-phase interface by the external stress imagining in Figure 6b. As shown in Figure 4d,e (with arrows), lots of voids and cracks can be clearly observed. It is hypothesized that the micro-cracks were generated during the impact test and then expanded completely so that the micro-particles are exposed out of the PP matrix. 

Therefore, the yield strength is reflected by the strength of interphase $\sigma_{g}$ instead of $\sigma_{f}$ (g denotes the interphase). The CaCO_3_ fillers replaced the space of PP in the composites and suffered the stress which only reached up to $\sigma_{g}$ then the micro-crack was generated. Hence, according to the Voigt model (the rule of mixtures) and considering the hybrid effect h, the yield strength of the composite $\bar{\sigma }$ is described by

(11) $$\bar{\sigma }=\left(1-v_{f}\right)\sigma_{m}+v_{f}\sigma_{g}+h\left(v_{f}\right),$$

where $\sigma_{m}$ is the yield strength of PP matrix. The yield strength of the composite is a nice linear relation whose slope is a constant K according to the experimental results as shown in Figure 7b. Thus, $dh/dv_{f}=K-\sigma_{g}+\sigma_{m}=\sigma_{h}$, which is constant for CaCO_3_ contents. Combining with Equation (11) and ${\bar{\sigma }|}_{v_{f}=0}=\sigma_{m}$, the yield strength is characterized by

(12) $$\bar{\sigma }=\sigma_{m}+\left(\sigma_{f}^{\prime }-\sigma_{m}\right)v_{f},$$

where $\sigma_{f}^{\prime }=\sigma_{g}+\sigma_{h}$ is termed by modified yield strength of CaCO_3_. The fitting results of Equation (12) are shown in Table 2. It indicates that the enhancement of a composite should make sure $\sigma_{f}^{\prime }>\sigma_{m}$, which is benefited by a well-dispersed effect and strong interfacial strength. $\sigma_{f}^{\prime }$ is an evaluation index of the interactional strength between matrix and filler. In addition, if there is a negligible hybrid effect with different contents, the modified yield strength is the same as the yield strength of interphase $\sigma_{f}^{\prime }=\sigma_{g}$. Of course, the $\sigma_{f}^{\prime }$ may be changed with different contents of fillers in other cases reflected in $\sigma_{h}$, where the yield strength of composites $\bar{\sigma }$ is not a linear relation with contents $v_{f}$ anymore. Furthermore, in case of multifold fillers, regarding $\bar{\sigma }$ as $\sigma_{m}$ when a new filler was joined, Equation (12) can be extended as (Proven in Appendix C)

$$\bar{\sigma }=\sigma_{m}+\sum_{i=1}^{n}\left(\sigma_{fi}^{\prime }-\sigma_{m}\right)v_{fi},$$

where n is the kinds of fillers and $v_{m}+\sum_{i=1}^{n}v_{fi}=1$. $v_{fi}$ and $\sigma_{fi}^{\prime }$ are the volume fraction and modified yield strength of each kind of filler, respectively. $\sigma_{fi}^{\prime }$ is the evaluation index of the interactional strength between matrix and ith filler in condition of uniaxial tension or three-point bending.

## 5. Conclusions

In this work, the strain localization coefficient ${\bar{B}}_{f}$ is formulated by a tensor γ independent of filler-contents based on the only hypothesis that self-consistent and differential self-consistent methods have an identical strain localization relation in the same materials. Hence, a combined self-consistent method was introduced to estimate the effective properties of two-phase composites. Both anisotropic solution Equation (5) and isotropic solution Equation (8) of the combined method are given and discussed in detail, which are verified by comparing with the Mori–Tanaka method and with the FEA confirmed SP model, respectively. Isotropic solution Equation (8) with just one parameter $\gamma_{v}$ conforms well to the experimental data of polypropylene/calcium carbonate composites, which indicates an effective application of the combined self-consistent method to describe the modulus of a composite even for the irregular filler shapes or poor strength of interphase. Moreover, an evaluation index $\sigma_{f}^{\prime }$ of the interactional strength between matrix and fillers is proposed based on experiments, which provides a quantitative method to select different surface modifiers ($\sigma_{f}^{\prime }>\sigma_{m}$). In experiments, a hyper-dispersant (PEPA-*g*-PHS) was synthesized successfully to prepare PP/CaCO_3_ composites up to 70 wt % of filler-content with dispersion confirmed by SEM, where the dosage of PEPA-*g*-PHS was only 5 wt % of the CaCO_3_ contents. 

## Figures and Tables

**Table 1:** The values of fitting parameters of two isotropic models.

Parameters	Approximate Model		Accurate Model		
	$\gamma_{v}$	Adj. *R*^2^	$\gamma_{1}$	$\gamma_{2}$	Adj. *R*^2^
Tension	0.0057	0.9768	0.0069	0.0073	0.9886
Bending	0.0149	0.9815	0.1408	0.0170	0.9748

**Table 2:** The value of fitting parameter of model.

Parameters	$\sigma_{f}^{\prime }$ (MPa)	Adj. *R*^2^
Tension	24.7957	0.9881
Bending	31.4176	0.9787

**Figure A1:**
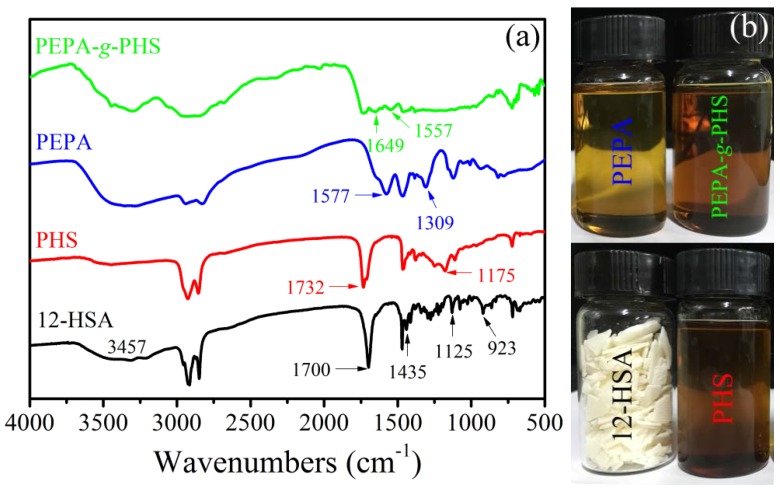
(**a**) FT-IR spectra and (**b**) Optical photographs of 12-HSA, PHS, PEPA, and PEPA-*g*-PHS.

**Figure A2:**
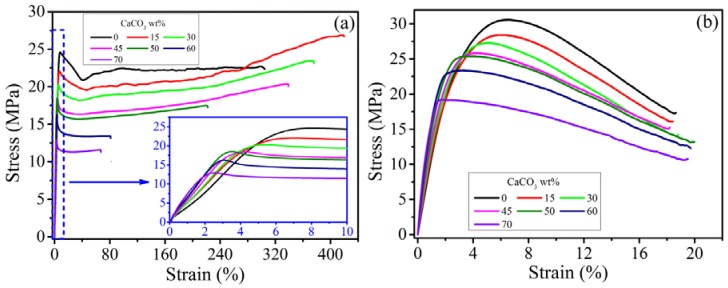
Typical (**a**) tensile and (**b**) bending stress-strain curves of different contents PP/CaCO_3_ composites.

**Figure A3:**
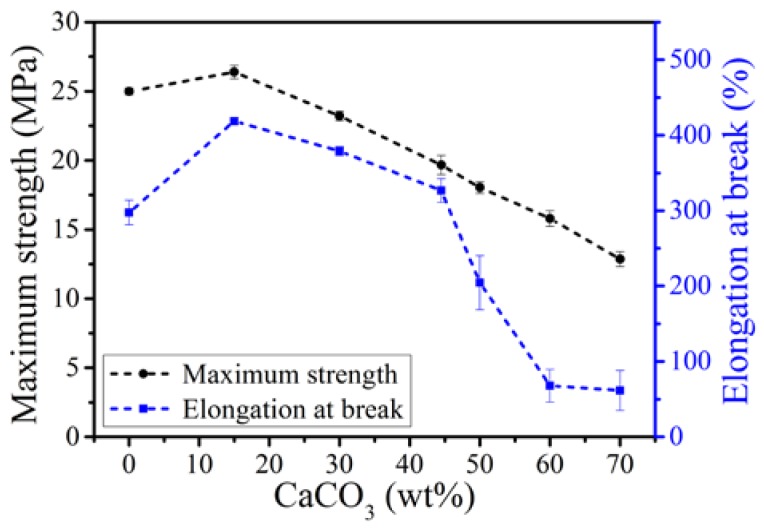
The maximum strength and elongation at break in uniaxial tension.

## Appendix A

Fourier-transform infrared spectra (FT-IR) were record on a Nicolet 470 spectrophotometer using KBr pellets at room temperature over the range of FT-IR 4000–500 cm^−1^. The viscosity of PEPA-*g*-PHS was measured using a Tu-4 cup (LND-1). Leveling the Tu-4 cup and keeping in thermostat with 25 °C, it was filled up with the PEPA-*g*-PHS without bubbles. The time measurement was started at the moment of turning on valve and ended at the moment of filament line broken.

The FT-IR spectra of 12-HSA, PHS, PEPA, and PEPA-*g*-PHS were shown in Figure A1a. There are the characteristic absorption bands of the 12-HSA at 1700 cm^−1^ (C=O stretching), 1125 cm^−1^ (C–O stretching), and 3457 cm^−1^ (O–H stretching) in spectrum. In addition, the in-plane and out-plane bending vibration of hydroxyl group (–OH) at the peaks of 1435 and 923 cm^−1^ are presented, respectively. The relevant peaks of the hydroxyl groups of 12-HSA at about 3457, 1435, and 923 cm^−1^ disappear in the spectrum of PHS. As well as the peaks of C=O stretching and C–O stretching of ester group at 1732 and 1175 cm^−1^ appear in the spectrum of PHS, respectively. It indicated that the esterification reaction among 12-HSA itself was sufficient and ester group was generated. PEPA is characterized by the absorption bands at 1577 cm^−1^ (–NH– stretching) and 1309 cm^−1^ (–NH_2_ stretching). In the spectrum of PEPA-*g*-PHS, the relevant peaks of the amino group of PEPA at 1577 and 1309 cm^−1^ disappear while the peak of C=O stretching of acylamino at 1649 cm^−1^ and N–H stretching of acylamino at 1557 cm^−1^ are presented. It indicated that the primary amine and secondary amine were reacted and acylamino group was generated. Hence, the results indicated that PEPA-*g*-PHS was synthesized successfully. Meanwhile, the optical photographs of 12-HSA, PHS, PEPA, and PEPA-*g*-PHS are shown in the Figure A1b. And results of Tu-4 cup viscosities of ingredients are shown in Table A1. The viscosities of ingredients should be converted according to the outflow time that is constrained within 150 s. The time of outflow of PEPA-*g*-PHS is far beyond 150 s. Thus, there is just a qualitative realization about the viscosity of PEPA-*g*-PHS according to the outflow time of PEPA, PHS and PEPA-*g*-PHS, which is obviously reflected in its order of magnitude.

**Table A1:** The Tu-4 cup viscosities of PEPA, PHS and PEPA-*g*-PHS.

Ingredients	PEPA	PHS	PEPA-*g*-PHS
Time (s)	75.3	628.7	4471.5

## Appendix B

Figure A2 shows the typical tensile and bending stress-strain curves of different content PP/CaCO_3_ composites. Figure A3 exhibits the influence of CaCO_3_ contents to the maximum strength and the elongation at break of composites. Maximum strength was obtained from the maximum value of yield strength and breaking strength.

## Appendix C

The $V_{fi}$ and $\sigma_{fi}^{\prime }$ denote the volume fraction and modified strength of a new joined filler, respectively. As well as ${\bar{\sigma }}_{i}$ is the strength of the generated composite (the new composite) while ${\bar{\sigma }}_{i-1}$ is of the old one. According to Equation (12), we obtain

$${\bar{\sigma }}_{i}={\bar{\sigma }}_{i-1}+\left(\sigma_{fi}^{\prime }-{\bar{\sigma }}_{i-1}\right)V_{fi}.$$

This is a recursion formula started with ${\bar{\sigma }}_{1}=\sigma_{m}+\left(\sigma_{f1}^{\prime }-\sigma_{m}\right)V_{f1}$. Therefore

$${\bar{\sigma }}_{n}=\sigma_{m}\prod_{i=1}^{n}\left(1-V_{fi}\right)+\sum_{j=2}^{n}\sigma_{f\left(j-1\right)}^{\prime }V_{f\left(j-1\right)}\prod_{i=j}^{n}\left(1-V_{fi}\right)+\sigma_{fn}^{\prime }V_{fn},$$

and mark

$$v_{f\left(j-1\right)}=V_{f\left(j-1\right)}\prod_{i=j}^{n}\left(1-V_{fi}\right),\;v_{fn}=V_{fn},$$

where $v_{fj}$ (j=1,2,…,n) is exactly right at the volume fraction of the jth filler in the final composite that contains n kinds of fillers. Meanwhile

$$v_{f\left(j-1\right)}=\prod_{i=j}^{n}\left(1-V_{fi}\right)-\prod_{i=j-1}^{n}\left(1-V_{fi}\right).$$

Thus

$$\sum_{j=2}^{n}v_{f\left(j-1\right)}=\left(1-V_{fn}\right)-\prod_{i=1}^{n}\left(1-V_{fi}\right),$$

$${\bar{\sigma }}_{n}=\sigma_{m}+\sum_{j=2}^{n}\left[\sigma_{f\left(j-1\right)}^{\prime }-\sigma_{m}\right]v_{f\left(j-1\right)}+\left(\sigma_{fn}^{\prime }-\sigma_{m}\right)v_{fn}.$$

Thereby

$$\bar{\sigma }=\sigma_{m}+\sum_{i=1}^{n}\left(\sigma_{fi}^{\prime }-\sigma_{m}\right)v_{fi},\;v_{m}+\sum_{i=1}^{n}v_{fi}=1.$$
